# Central venous catheters: incidence and predictive factors of venous thrombosis 

**DOI:** 10.5414/CN108347

**Published:** 2015-05-22

**Authors:** Mary Hammes, Amishi Desai, Shravani Pasupneti, John Kress, Brian Funaki, Sydeaka Watson, Jean Herlitz, Jane Hines

**Affiliations:** 1 Section of Nephrology,; 2 University of Chicago,; 3 Section of Pulmonary and Critical Care, Department of Medicine,; 4 Department of Radiology, and; 5 Department of Health Studies, Biostatistics Laboratory, University of Chicago, Chicago, IL, USA

**Keywords:** catheter, fistula, renal failure, thrombosis

## Abstract

Aims: Central venous catheter access in an acute setting can be a challenge given underlying disease and risk for venous thrombosis. Peripherally inserted central venous catheters (PICCs) are commonly placed but limit sites for fistula creation in patients with chronic renal failure (CKD). The aim of this study is to determine the incidence of venous thrombosis from small bore internal jugular (SBIJ) and PICC line placement. This investigation identifies populations of patients who may not be ideal candidates for a PICC and highlights the importance of peripheral vein preservation in patients with renal failure. Materials and methods: A venous Doppler ultrasound was performed at the time of SBIJ insertion and removal to evaluate for thrombosis in the internal jugular vein. Data was collected pre- and post-intervention to ascertain if increased vein preservation knowledge amongst the healthcare team led to less use of PICCs. Demographic factors were collected in the SBIJ and PICC groups and risk factor analysis was completed. Results: 1,122 subjects had PICC placement and 23 had SBIJ placement. The incidence of thrombosis in the PICC group was 10%. One patient with an SBIJ had evidence of central vein thrombosis when the catheter was removed. Univariate and multivariate analysis demonstrated a history of transplant, and the indication of total parenteral nutrition was associated with thrombosis (p < 0.001). The decrease in PICCs placed in patients with CKD 6 months before and after intervention was significant (p < 0.05). Conclusions: There are subsets of patients with high risk for thrombosis who may not be ideal candidates for a PICC.

## Introduction 

The arteriovenous fistula (AVF) remains the preferred access to provide hemodialysis to patients with end-stage renal disease (ESRD). Regulatory agencies including the Renal Network and Center for Medicare and Medicaid Services (CMS) recommend permanent AVF access as patient outcomes are improved when a fistula is used for dialysis. When compared to central venous catheters, complications with fistulas and grafts are much lower [1, 2]. However, the success of a fistula or graft is dependent on underlying preservation of central and peripheral veins prior to the patient starting on dialysis. It is therefore imperative that clinical guidelines recommending the use of an AVF are followed to preserve veins in patients with CKD and ESRD. 

Obstacles to venous access placement include previous damage from phlebotomy, and peripherally inserted central catheters (PICCs). A PICC is a catheter placed in a peripheral cephalic or basilic vein used to deliver chemotherapy, antibiotics or total parenteral nutrition (TPN). They are often left in place for weeks or months. Given the relative ease of insertion, PICCs have become commonplace in hospitals across the country [3, 4]. 

Although PICC lines are considered safe with a low incidence of complications, placement can result in phlebitis, stenosis or thrombosis of the involved veins [5, 6]. Previous studies have shown that the risk of PICC associated deep venous thrombosis is comparable to rates seen in individuals with hypercoagulable states [7]. Additional factors leading to increased rates of thrombosis include: catheter diameter, history of previous thromboembolism, renal failure, and surgery for longer than 1 hour [8, 9]. Once the vein is damaged, sclerosis may result and prevent future use of the vein for hemodialysis fistula access. 

**Figure 1.**
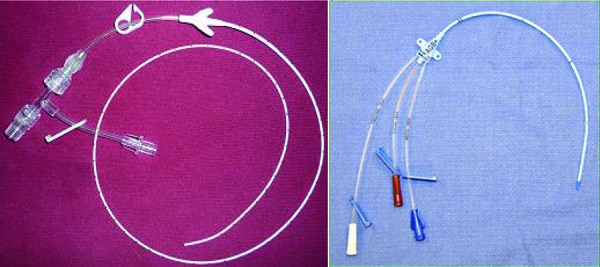
PICC (left panel) and small bore IJ catheter (right panel).

Current guidelines suggest that no peripheral vein should be considered “expendable” in the high risk chronic kidney disease (CKD) and renal transplant population [10]. Instead, a small bore internal jugular catheter (SBIJ) is the preferred access in this subgroup of patients [11, 12]. Compared to a PICC, the SBIJ is a shorter catheter inserted in the internal jugular vein (IJV), with a smaller diameter (5 – 6 French) than conventional catheters (7 – 9 French) placed in this location (Figure 1). However, the thrombosis rate after placement of such small-bore catheters has never been prospectively reviewed. 

The aim of the current study is to determine the incidence of central or peripheral venous thrombosis from SBIJ and PICC line placement. The intent was that by recommending SBIJ placement in patients with CKD in a prospective trial, the practice pattern of placing PICCs in patients with CKD would change. A secondary aim is to determine if variables such as renal function, diabetes, hypertension, cancer, transplant or a history of atherosclerosis contribute to the risk of thrombosis when PICCs are placed. To our knowledge, these data provide the first evidence of the incidence of thrombotic complications from placement of an SBIJ venous access. 

## Methods and materials 

The study was conducted at a single, large, university-based medical center from September 1, 2010 through December 31, 2011. This study was submitted to, and approved by the Institutional Review Board at the University of Chicago (Protocol # 10-145-A) with adherence to the Declaration of Helsinki. Written consent was obtained in all subjects undergoing SBIJ access placement. Subjects were screened for inclusion by daily review of a Procedure Service Log for PICC line insertion by a Nephrologist. Patients with a history of CKD Stage-3, estimated glomerular filtration rate (eGFR) less than 60 mL/min for at least three months prior to admission, ESRD, or a renal transplant were considered for inclusion in the study. The patient was further evaluated for intravenous (IV) access needs by contacting the patient’s primary care service. If the patient required IV access with a central venous catheter and met one of the aforementioned criteria without a contra-indication for central venous access, the Nephrologist would advise the primary service to order an SBIJ to be placed by Interventional Radiology in substitution for a PICC access. The Nephrologist then consented all patients meeting inclusion criteria, explaining the risk/benefit advantage to use of SBIJ access in patients with CKD. Contraindications to enrollment in the study included anti-coagulation which could not be reversed (i.e., warfarin, therapeutic enoxaparin), severe thrombocytopenia (platelet < 30,000), and any current vasoactive therapy. Written consent was obtained prior to catheter access by Interventional Radiology. 

PICC placement was performed solely by the procedure service at the bedside with a 5 French catheter (Boston Scientific, Inc., Marlborough, MA, USA) using ultrasound guidance. SBIJ placement was performed solely by an Interventional Radiologist with either a single lumen 5 French or double lumen 6 French small bore catheter (C. R. Bard, Inc., Murray Hill, NJ, USA). At time of placement, a real time Doppler ultrasound image of the IJV was taken to determine patency, vein dimensions, and ensure the vessel was free of stenosis and thrombosis. A guide wire was advanced into the SVC under fluoroscopic guidance. Using blunt dissection, a subcutaneous tunnel was created to allow placement of the catheter. Spot thoracic radiographs were performed to ensure the tip of the catheter was in the right atrium or superior vena cava. The catheter was flushed with saline and secured to the skin with non-absorbable suture. Once it was time for catheter removal, patients again returned to Interventional Radiology and underwent an ultrasound to evaluate for presence of thrombus and measure vein diameter just prior to removal. 

PICC data was gathered by conducting a chart review from September 1, 2010 through December 31, 2011. Patients who received a PICC during the study period were determined by reviewing procedure log billing sheets. A separate IRB was generated for this portion of data collection with waived consent (Protocol # 12-1889) with adherence to the Declaration of Helsinki. Patient demographics and past history including diabetes, hypertension, peripheral vascular disease (PVD), coronary artery disease (CAD), transplant, history of cancer, and CKD were obtained from paper and electronic medical records for all patients included in the study. A patient was considered to have CAD if they had a prior history of myocardial infarction or cardiac catheterization performed showing significant coronary disease. A patient was considered to have PVD if they had a prior history of amputation from ischemic disease or a vascular study showing ankle-brachial index less than 0.9. A patient was counted as a transplant if they had received a hematopoietic or solid organ transplant. Indications for venous access and duration of catheter were recorded. A thrombotic event was defined as evidence of peripheral or central venous thrombosis by an Upper Extremity Venous Duplex Exam, CT of the chest or an echocardiogram after PICC or SBIJ placement. If a patient had a thrombotic event evident on Venous Duplex it was counted as a central occlusion if thrombosis was evident in the internal jugular, brachiocephalic, subclavian or axillary vein; peripheral if thrombosis was evident in the cephalic, basilica, or brachial vein; or both if the thrombosis was evident in the central and peripheral veins. If a patient had a CT of the chest done and there was evidence of central vein thrombus or a pulmonary emboli, it was counted as a central thrombotic event. If a patient had an echocardiography done and a right atrial thrombus was evident, it was counted as central thrombotic event. 

Patient age, BMI, and duration days associated with first catheter placement were summarized by mean and standard deviation. Wilcoxon rank sum test was used to compare these baseline characteristics in patients who did or did not experience thrombosis in any of his/her PICC placements. Counts and percentages were used to summarize categorical variables associated with first catheter placement; Fisher’s exact test was used to statistically test for an association between each categorical variable and thrombosis. p-values in all univariate analyses were adjusted according to the Benjamini-Hochberg method and assessed at a 5% false discovery rate. Univariate analyses among subjects with PICC’s were used to build several regression models of two varieties. Logistic regression models of overall thrombosis status were adjusted for variable values at time of first catheter placement. Generalized estimating equations (GEE) were used to fit logistic regression models of all thrombosis outcome that were adjusted for covariate values in all PICC placements evaluated for a single patient. Each model included at least one variable shown to be related to thrombosis and excluded correlated variables identified in pairwise analyses. p-values were assessed at the 5% significance level. All statistical analyses were performed in R version 3.0.2 http://www.r-project.org/. 

## Results 

### Patient characteristics 

There were 1402 PICC lines placed during the study period in 1,122 subjects. Of these, 114 patients (10%) had evidence of documented central or peripheral vein thrombosis on at least one catheter placement as evident by venous Doppler, CT, or echocardiography. Of these, 44 (38%) patients had evidence of a central thrombotic event, 35 (31%) had a peripheral thrombotic event, and 35 (31%) had a thrombotic event which included both central and peripheral veins. Table 1 details the clinical characteristics of those individuals with or without thrombosis incidence recorded for any PICC placed. All groups had relatively similar age, proportion of male subjects, and average BMI. There were only 18.4% of patients with CKD who had PICC lines placed. The average duration of days of catheter placement was greater in the thrombotic group when compared to the nonthrombotic group, but did not reach statistical significance even when adjusting for the various indications (p > 0.05). The subjects who experienced a thrombotic event differed significantly with respect to percentages of patients receiving transplant and receiving PICC’s for various indications. In particular, patients whose underlying medical conditions merited the need for total parenteral nutrition (TPN) were more likely to experience thrombosis events whereas those who needed antibiotics had a lower risk of thrombosis. There were 21 subjects with a transplant who developed thrombosis after a PICC placement. 13 had received an autologous stem cell transplant, 6 were heart transplant recipients, 1 had received a liver transplant, and 1 a renal transplant. 

Clinical characteristics of subjects enrolled in the study that had SBIJs are shown in Table 2. 28 subjects were consented for the study with 26 catheters placed in 23 completing the study. A right IJ catheter was placed in 25 subjects, a left catheter in 1 subject. A single lumen catheter was placed in 4 subjects and a double lumen catheter was placed in 22 subjects. 

There was only 1 subject in the SBIJ group who was found to have venous thrombosis at the time of catheter removal (p = 0.53 compared to standard PICC intervention). This subject was a 43-year-old male with a history of CAD, PVD, and hypertension with right dual-lumen SBIJ placed for antibiotics for 9 days. The patient had a BMI of 54.8, which was significantly higher when compared to other subjects in this study group (28.3 ± 9.1). 

### Multivariate analysis 

Analysis of pairwise relationships between covariates revealed evidence of significant correlation between indication and transplant status. Thus, we considered separate models of thrombosis status that included either transplant or indication as a covariate. Relative to antibiotics, patients with an indication of chemotherapy, other, and TPN were at higher risk of thrombosis than those whose indication was for antibiotics (Table 3). The increased risk for subjects with immunosuppression indication was not significant. Subjects who received a transplant were also at a significantly higher risk (Table 4). Findings in the GEE analysis were similar to univariate and multivariate analysis, as indication of TPN, other and chemotherapy were significant. In the GEE model, CAD was found to be a significant risk factor for thrombosis; however, there was no evidence of a significant relationship between transplant and thrombosis risk. A regression model was done which examined the association between renal function and thrombosis after adjusting for transplant (the only factor in the study that was significantly associated with thrombosis but not with renal function). There was no significant association between thrombosis and renal function (p = 0.35). 

### Intervention analysis 

There were 580 PICC’s placed six months prior to the study (March 1, 2010 – August 31, 2010) by the procedure service. Of these 108 (18.6%) had evidence of stage III or greater CKD or a renal transplant. Of the 630 PICC’s placed 6 months after the study (January 1, 2012 – June 20, 2012), 85 (13.5%) were in patients with stage III or greater CKD or had a renal transplant. The decrease in PICC’s placed in patients with CKD from 18.6% (6 months before) to 13.5% (6 months after) was significant (p < 0.05). 

## Discussion 

Thrombotic complications are common after PICC placement. Our data matched the previous published literature regarding thrombosis rate from PICCs [4, 8, 14, 15]. The thrombotic rate, as measured by thrombus evident at time of catheter removal, was minimal in patients with renal failure who received an SBIJ. In our patient population, the risk of thrombosis after PICC placement was increased in patients who had received a transplant or those who received a PICC for administration of TPN. 

Patients with a transplant had a higher risk of thrombosis in our series. Most of these subjects had a recent stem cell transplant. Previous reports using PICC’s in patients who receive autologous stem cell transplant have concluded that the PICC is a safe and effective alternative to conventional central vein catheters [16]. Others have shown a higher rate of complications including infection and thrombosis in onco-hematological patients [17]. Chemotherapy has also been shown to increase the risk of venous thrombosis and may have been a contributing factor in this patient group [18]. TPN has been identified as a risk factor for venous thrombosis in previous studies [9]. Patients receiving PICC’s for antibiotic administration may have had a lower incidence of thrombosis as these patients may have had a single lumen catheter placed when compared to those patients receiving TPN. 

Risk factors that predispose to central venous catheter-associated thromboses have been identified in multiple studies in the past. It has been shown that an increased diameter of the catheter used is associated with an increased risk of thrombosis [6]. Another less commonly cited factor is the position of the catheter tip. Ideally, the catheter tip should terminate at the high flow SVC/RA junction. Previous studies have shown a higher risk of thrombosis for central venous catheters terminating in the brachiocephalic vein or cranial superior vena cava when compared to those placed at the appropriate position as above [19]. The duration of the catheter placement has also been shown to be associated with an increased risk of thrombosis [20]. The number of lumens in the catheter (dual vs. single) has been identified as a contributing risk factor to thrombosis and infection from PICC placement [8]. Given the retrospective nature of data collection in the PICC group in our study, we were not able to identify some of these important technical properties and exact location of the PICC placed. 

Efforts to prevent PICCs in patients with renal failure and transplant should be made as they lead to sclerosis of the peripheral vein, preventing use for hemodialysis in the future [12]. There are only two superficial venous systems in each arm, the cephalic and the basilic. PICCs are often inserted into the basilic system. Vein injury anywhere along the length of the catheter, is considered a primary initiating event for catheter-related thrombosis which may make the entire extremity useless for future placement of an AVF [21, 22]. Furthermore, associated thrombi pose problems both in terms of short term management of line dysfunction due to the clot, and if not detected early, may eventually lead to propagation of the clot to the catheter tip or into the peripheral vein [23]. We showed that PICC placement is a very common procedure with an average of over 100 PICC’s placed per month at a busy university center. Of these, many were placed in patients with CKD. By changing practice (notifying the procedure service of the need to prevent PICC placement in this high risk population), we decreased PICC placement from 18.6% to 13.5%. 

There are a number of key limitations to our study. Among these is the small sample size of patients who received SBIJ catheters. As the short term risk of thrombosis was shown to be minimal and we had changed practice patterns, this arm of the study was terminated early. Another limitation is that the study did not account for inherited hyper-coagulable states in either group, a factor that may confound the results. A significant portion of subjects were lost to follow up or had incomplete medical records, though all attempts were made to ensure a complete database. A thrombotic event, was determined from a retrospective review of imaging procedures that diagnosed a thrombotic event. These diagnostic exams were performed after a clinical event in most cases. Each procedure (Venous duplex, CT, or echocardiogram) has their own inherent error and some patients may have had sub-clinical thrombosis which was not detected. A prospective trial using similar diagnostic methods in all patients is needed to validate our findings. Longer term follow up perhaps at 3 – 6 months with Doppler analysis to determine the incidence of thrombosis would also be of interest and will be studied in the future. IJV thrombosis is often asymptomatic and therefore not detected. The incidence of pulmonary emboli related to IJV thrombosis is significant and likely underreported [23]. Although we assert that our interventional study led to less PICC line use, it is certainly plausible that there was just less need for PICC placement in the post-6 month follow up period vs. the pre-intervention period. 

In summary, our study identifies a sub-group of patients, who may not be ideal candidates for PICC placement. We need to carefully identify those patients in need of venous access including: the indication, underlying medical history, and type of catheter required. If a short term central venous access is needed for a patient in these high risk subgroups, it should be placed only after a multidisciplinary discussion between the primary service, Nephrologist (if the patient has CKD) and the access team in order to avoid thrombosis, choosing a site that best preserves future dialysis access sites. Future perspective trials are needed to validate our findings and confirm the type of catheter recommended for high risk patient sub-groups. 

## Conflict of interest 

None. 

## Acknowledgments 

This publication was made possible in part by the National Institute of Diabetes and Digestive Diseases (NIDDK) and the National Institutes of Health (NIH) under award number RO1DK090769. The content is solely the responsibility of the authors and does not necessarily represent the official views of the NIDDK or the NIH. 

**Table 1.** Demographics of PICC group.

	No thrombosis	Thrombosis	p-value^*^
n	1,007	114	
Average age (years)	56.9 ± 18.4	58.0 ± 16.42	0.78
Men (%)	503 (50)	61 (53.5)	0.78
Average BMI	27.9 ± 10.5	27.5 ± 9.1	0.86
Indications for PICC			
Antibiotics (%)	343 (34.1)	13 (11.4)	< 0.001^*^
Immunosuppressant (%)	6 (0.6)	1 (0.9)	0.78
Chemotherapy (%)	182 (18.1)	23 (20.2)	0.78
TPN (%)	44 (4.4)	24 (21.1)	< 0.001*
Other (%)	432 (42.9)	53 (46.5)	0.78
Renal failure			0.78
AKI (%)	137 (13.6)	12 (10.5)	
CKD (%)	186 (18.5)	20 (17.5)	
Neither	684 (67.9)	82 (71.9)	
Diabetes (%)	223 (22.1)	29 (25.4)	0.78
HTN (%)	383 (38)	43 (37.7)	1
PVD (%)	48 (4.8)	4(3.5)	0.86
CAD (%)	139(13.8)	26 (22.8)	0.06
Transplant (%)	86 (8.5)	21 (18.4)	< 0.001^*^
Cancer (%)	327 (32.5)	45 (39.5)	0.4
Duration of catheter (days)	88.1 ± 163.1	116.4 ± 187.9	0.78
Antibiotics	133.1 ± 194.7	98.7 ± 110.0	0.91
Immunosuppressant	256.7 ± 417.7	23 ± NA	–
Chemotherapy	50.3 ± 122.2	132.3 ± 214.8	0.47
TPN	87.0 ± 100.2	47.0 ± 51.5	0.13
Other	79.4 ± 156.1	146.8 ± 223.6	0.10

Values are means ± SE or count (%) as appropriate. BMI = body mass index; PICC = peripherally inserted central catheter; TPN = total parenteral nutrition; AKI = acute kidney injury; CKD = chronic kidney injury; HTN = hypertension; PVD = peripheral vascular disease; CAD = coronary artery disease. *Benjamini-Hochberg method of p-value adjustment was used to control the false discovery rate at 5%.

**Table 2.** Demographics of SBIJ group. Values are means ± SE or count (%) as appropriate.

	Nonthrombotic	Thrombotic
n	22	1
Average age (years)	48.4 ± 13.8	43
Men (%)	10 (45.5)	1 (100)
Average BMI	28.3 ± 9.1	54.8
Indications for SBIJ		
Antibiotics (%)	9 (40.9%)	1 (100%)
Immunosuppressant (%)	12 (54.5%)	
Chemotherapy (%)	0 (0%)	
TPN (%)	1 (4.5%)	
Other (%)	0 (0%)	
Diabetes	15 (54.5%)	+
HTN	17 (77.3%)	+
PVD	3 (13.6%)	+
CAD	6 (27.3%)	+
Transplant	18 (81.8%)	–
Duration of catheter (days)	12.9 ± 8.5	9.0
Antibiotics	19.3 ± 9.1	9.0
Immunosuppressant	8.8 ± 4.4	
Chemotherapy		
TPN	4.0 ± NA	
Other		

BMI = body mass index; SBIJ = small bore internal jugular catheter; TPN = total parenteral nutrition; HTN = hypertension; PVD = peripheral vascular disease; CAD = coronary artery disease.

**Table 3.** Multiple regression model of thrombosis adjusted for indication relative to antibiotics.

	Odds ratio	95% Confidence interval	p-value
Chemotherapy	3.00	(1.1, 8.2)	0.031
Immunosuppression	6.69	(0.5, 85.0)	0.14
Other	3.00	(1.2, 7.5)	0.016*
TPN	13.46	(4.5, 40.0)	< 0.001*
Age (years)	1.00	(1.0, 1.0)	0.71
Duration (days)	1.00	(1.0, 1.0)	0.081

Values expressed as odds ratio with 95% confidence interval as appropriate. TPN = total peripheral nutrition. *Benjamini-Hochberg method of p-value adjustment was used to control the false discovery rate at 5%.

**Table 4.** Multiple regression model of thrombosis adjusted for transplant relative to antibiotics. Values expressed as odds ratio with 95% confidence interval as appropriate. CAD = coronary artery disease.

	Odds ratio	95% confidence interval	p-value
Transplant	11.02	(5.9, 21.0)	0.009*
Duration (days)	1.00	(1.0, 1.0)	0.091
CAD	1.93	(0.9, 4.1)	0.083

*Benjamini-Hochberg method of p-value adjustment was used to control the false discovery rate at 5%.
